# Factors associated with SARS-CoV-2 RNAemia development at COVID-19 diagnosis

**DOI:** 10.1371/journal.pone.0330495

**Published:** 2025-08-21

**Authors:** Javier Martín-Escolano, Ana Ruiz-Molina, Cristina Rodríguez-Urbistondo, Carmen Infante-Domínguez, Gabriela Abelenda-Alonso, Gorane Euba, Zaira R. Palacios-Baena, Francisco Arnaiz-Revillas, Jorge Alba, Alexander Rombauts, Regino J. Rodríguez-Álvarez, Natalia Maldonado, Claudia González-Rico, Sonia Santibañez, Marta Carretero-Ledesma, Laura Merino, Jordi Carratalá, Ane Josune Goikoetxea-Agirre, Marta Fernández-Martínez, José Antonio Oteo, María Carmen Fariñas, Jesús Rodríguez-Baño, José Miguel Cisneros, Belén Gutiérrez-Gutiérrez, Rocío Álvarez-Marín, Manuela Aguilar-Guisado, Sonsoles Salto-Alejandre, Elisa Cordero, Jerónimo Pachón, Javier Sánchez-Céspedes

**Affiliations:** 1 Clinical Unit of Infectious Diseases, Microbiology and Parasitology, Instituto de Biomedicina de Sevilla (IBiS), Virgen del Rocío University Hospital/CSIC/University of Seville, Seville, Spain; 2 CIBER de Enfermedades Infecciosas, Instituto de Salud Carlos III (CIBERINFEC, ISCIII), Madrid, Spain; 3 Service of Infectious Diseases, Bellvitge University Hospital, Bellvitge Biomedical Research Institute (IDIBELL), University of Barcelona, Hospitalet de Llobregat, Barcelona, Spain; 4 Department of Infectious Diseases, Hospital Universitario Cruces, Bizkaia, Spain; 5 Clinical Unit of Infectious Diseases and Microbiology, Virgen Macarena University Hospital, Instituto de Biomedicina de Sevilla (IBiS)/CSIC/University of Seville, Seville, Spain; 6 Service of Infectious Diseases, Marqués de Valdecilla University Hospital, Marqués de Valdecilla-IDIVAL, University of Cantabria, Santander, Spain; 7 Department of Infectious Diseases, San Pedro-CIBIR University Hospital, Logroño, La Rioja, Spain; 8 Department of Medicine, School of Medicine, University of Seville, Seville, Spain; 9 Instituto de Biomedicina de Sevilla (IBiS), Virgen del Rocío University Hospital/CSIC/University of Seville, Seville, Spain; Children's National Hospital, George Washington University, UNITED STATES OF AMERICA

## Abstract

**Objectives:**

SARS-CoV-2 RNAemia at diagnosis is associated with mortality. The aims were to identify factors associated with the development of RNAemia.

**Methods:**

Multicenter COVID-19 cohort study was conducted between January 2020 and May 2023. Demographics, chronic underlying diseases, symptoms and signs, analytical and radiological variables, cytokines, and neutralizing antibodies were evaluated on admission. RNAemia was the primary endpoint.

**Results:**

We included 1011 patients, 392 (38.8%) immunocompromised and 619 (61.2%) immunocompetent. RNAemia occurred in 49.7% and 18.7% (p < 0.001), respectively, being independently associated with 30-day all-cause mortality. In immunocompromised patients, factors independently associated with RNAemia were Alpha and Omicron VOC periods (OR: 1.95 [1.01–3.79]), pneumonia (OR: 1.96 [1.10–3.50]), LDH > 300 UI/L (OR: 1.64 [1.02–2.63]) and neutralizing antibodies absence (OR: 2.51 [1.57–4.00]). In immunocompetent patients, the factors associated with RNAemia were Delta and Omicron VOC periods (OR: 2.27 [1.46–3.52]), lymphocyte count < 1000/µL (OR: 1.81 [1.16–2.80]) and LDH levels > 300 IU/L (OR: 3.99 [2.51–6.36]).

**Conclusions:**

Immunodeficiency almost tripled SARS-CoV-2 RNAemia. Omicron VOC period, LDH as inflammatory biomarker, and a lower immune response in all patients, neutralizing antibodies absence in immunocompromised and lymphopenia in immunocompetent, and pneumonia in immunocompromised patients were associated with RNAemia.

## Introduction

As of early June 2025, COVID-19 has caused hundreds of millions of infections and millions of deaths worldwide [1]. This pandemic has triggered an unprecedented global response from all health systems to advance epidemiological, virological, pathophysiological, clinical, and prognostic knowledge of severe acute respiratory syndrome coronavirus-2 (SARS-CoV-2) infection. Additionally, the availability of resources has allowed the development of new vaccines and antivirals in record time [2]. Currently, a high percentage of the world’s population is vaccinated against SARS-CoV-2 (67%) with a complete primary series, although there is high variability depending on the country [1]. In Spain, at the time this study was conducted, 79% of the population had been vaccinated using a complete primary series. COVID-19 shows great clinical variability mainly depending on the patient’s baseline condition, ranging from mild cases to critical illness with multiorgan failure and high mortality. Thus, much effort has been put into identifying quick and easy biomarkers of severity at the COVID-19 diagnosis, especially in patients requiring hospital-admission, to implement measures to improve patient management.

SARS-CoV-2 RNAemia, defined as the presence of viral RNA in the bloodstream, has emerged as an independent predictor of severe outcomes in COVID-19 patients [3], and support its role as a valid biomarker. Several reports with large cohorts have found a robust association between SARS-CoV-2 RNAemia and adverse clinical outcomes, including mortality [4–9]. Some of these studies, besides addressing the association of RNAemia with mortality, have also found an association among the presence of RNAemia and plasma viral load with underlying conditions, such as older age and comorbidities, CURB-65 score, or laboratory data such as baseline PaO_2_/FiO_2_, LDH, lymphopenia, C-reactive protein, D-dimer, ferritin, and cytokine (IL-6, IL-10, and IL-15) levels [4–6]. A study has showed an association of RNAemia at COVID-19 diagnosis with unfavorable outcome, defined as death and/or intensive care admission, in solid organ transplant recipients, besides with mortality in immunocompetent patients [9].

Other studies have shown an association between severe COVID-19 and immunological factors such as a lack of production of neutralizing antibodies [10–12] and low levels of IgG [13]. Some studies concluded that hospitalized patients have higher titers of neutralizing antibodies (NAb) than non-hospitalized patients [10,11], but the assayed samples were drawn at heterogeneous times after hospital admission; this may explain the discordant results with other studies evaluating the association of lack of NAb response with fatal outcomes, in which NAb and IgG levels remained in the recovered patients, irrespective of disease severity [12]. Additionally, an inverse association between RNAemia and a specific IgG antibody response has been shown in a cohort of patients with COVID-19 with diverse inclusion criteria, suggesting control of SARS-CoV-2 dissemination by the humoral immune response [13].

As vaccination rates have increased worldwide, the dynamics of SARS-CoV-2 transmission have undergone a profound shift [14]. The wide-scale deployment of vaccines, mainly mRNA-based formulations and vector vaccines, has demonstrated remarkable efficacy in preventing severe COVID-19 [2,14]; it has also raised questions about the mechanisms for the presence of SARS-CoV-2 RNAemia and its persistence, seen even in vaccinated patients, and its clinical impact. Unravelling the complexities of this association is essential, given the potential implications for the clinical management of these patients.

Previous studies have provided information on the association of RNAemia with the COVID-19 clinical outcome, mostly in immunocompetent patients, and contradictory information on the specific immune response and its association with RNAemia and COVID-19 severity. As other important issue, from the pandemic onset in 2020, SARS-CoV-2 has evolved to different variants of concern (VOC), showing genetic conservation and diversity, and with changes in virulence and pathogenicity [15]. In this context, the present study aimed to gain valuable insights into the association of host and virological factors, specific immune response and previous vaccination with the development of SARS-CoV-2 RNAemia at the onset of SARS-CoV-2 disease, both in immunocompromised and immunocompetent patients. The data obtained could generate the necessary knowledge to implement interventions aimed at preventing SARS-CoV-2 RNAemia and improving patient prognosis, especially in more vulnerable populations.

## Materials and methods

### Research design and participants

We conducted a prospective, multicenter, and non-interventional study, carried out in six Spanish hospitals, including both immunocompromised, from March 2020 to May 2023, and immunocompetent, from January 2020 to May 2022, patients. Inclusion criteria were: adult age (≥ 18 years), acute COVID-19 confirmed by a positive SARS-CoV-2 reverse transcription-PCR (RT-PCR) test from nasopharyngeal (NP) swabs, availability of blood samples to determine the presence of SARS-CoV-2 RNAemia in the first 24 h after diagnosis, and signed informed consent. Exclusion criteria included: asymptomatic patients, a life expectancy of less than one month, assessed by the physician judgment following the McCabe-Jackson criteria [16], and patients who declined to sign the informed consent form. This study was approved by the Ethics Committees of Virgen Rocío and Virgen del Macarena University Hospital (C.I. 0771-N-20 and 1683-N-21) and adhered to the Helsinki Declaration.

### Clinical data and sample acquisition

Immediately after enrolment, blood samples to determine the presence of SARS-CoV-2 RNAemia, cytokines, and specific immune response were obtained. Demographics, specific clinical signs and symptoms, and complementary data from standard microbiological, biochemical, and hematological studies were recorded for each patient using a protocol case report form. Disease duration was defined as the number of days from symptom onset to death or hospital discharge. Patients were attended following the decisions of the clinician teams in charge, based on local policies and standard clinical management practices, and were followed up until 30 days after COVID-19 diagnosis or until discharge or death, whichever occurred first. Disease severity at the time of COVID-19 diagnosis and the end of the episode was evaluated using the World Health Organization (WHO) Clinical Progression Scale [17].

### SARS-CoV-2 detection in NP swabs and blood

For SARS-CoV-2 RNA extraction from plasma samples (collected in EDTA tubes) and NP swabs, a MagNA Pure Compact Nucleic Acid Isolation Kit I (Roche Diagnostics GmbH, Mannheim, Germany) was used according to the manufacturer’s instructions. The RT-PCR assays employed the 2019-nCoV ValuPanel Reagents (LGC Genomics GmbH, Berlin, Germany) in addition to the GoTaq® Probe 1-Step RT-qPCR System (Promega Biotech Ibérica S.L, Madrid, Spain) and were run in a LightCycler 96 Instrument (Roche Diagnostics GmbH, Mannheim, Germany) following the manufacturer’s protocols, with a Ct detection limit of 42. The kit includes internal controls targeting two SARS-CoV-2 nucleocapsid genes and human RNase. SARS-CoV-2 RNAemia quantification was not performed because of it´s not different in immunocompromised *vs.* immunocompetent patients and its lack of association with unfavorable clinical outcome and/or mortality [9].

### Plasma levels of IFN-α and IFN-γ

Plasma samples were collected and stored at −80 °C before analysis. IFN-α (USCN Life Science & Technology Company, Missouri, TX, USA) and IFN-γ (RayBiotech, Norcross, GA, USA) levels were quantified using ELISA kits following the manufacturer’s instructions as described previously [18]. A Multiskan™ GO Microplate Spectrophotometre (Thermo Fisher Scientific, Inc., Waltham, MA, USA) was used to quantify optical density. Relative levels of IFN-α and IFN-γ were analysed using a log/log fit curve with GraphPad Prism 6. These assays, with limits of detection of 3 pg/mL for IFN-α and 2 pg/mL for IFN-γ, were performed in duplicate for each sample. The values used as a reference for healthy, uninfected adults are described in our previous article [9].

### Quantification of neutralizing antibodies

Neutralizing antibodies were evaluated using the viral microneutralization assay. Serum samples were collected from blood tubes with separator gel after centrifugation for 10 minutes at 3000 rpm and 4 °C. In a 96-well polystyrene microplate (Corning, New York, USA), serial dilutions of serum samples were prepared and 1.3 x 10^4^ TCID50/mL SARS-CoV-2 (Wuhan-Hu-1) spike pseudotyped lentivirus with a Green Fluorescent Protein reporter of infection (ATCC, Manassas, USA) was added. The mixture was added to microplates containing HEK293T cells expressing human ACE2 and TMPRSS2 (GeneCopoeia, Rockville, MD, USA) that were previously seeded. After incubation at 37 °C for 72 hours, microneutralization by serum was confirmed and quantified. The detection limit for the neutralizing antibodies titer was a 1/3 dilution.

### Statistical analysis

The primary endpoint was SARS-CoV-2 RNAemia at the time of COVID-19 diagnosis in both immunocompromised and immunocompetent patients. The associations between SARS-CoV-2 RNAemia and clinical conditions, initial symptoms, and immune and inflammatory variables were also evaluated. Descriptive statistics were presented as frequencies (%) or medians with interquartile ranges (IQRs). Continuous variables such as age, Charlson comorbidity index, oxygen saturation (SpO_2_), CURB-65, WHO clinical progression scale, lymphocytes, platelets, creatinine, C-reactive protein, D-dimer, and LDH were dichotomized based on their association with mortality [19]. The study was categorized into three periods depending on the predominant SARS-CoV-2 variant of concern (VOC) in Spain [20]: i) Alpha from January 2020 to June 27th, 2021; ii) Delta from June 28th to December 19th, 2021; and iii) Omicron from December 20th, 2021 to May 2023. Univariate analysis was conducted to assess the association between the variables and SARS-CoV-2 RNAemia at the time of COVID-19 diagnosis. Statistical tests for between-group comparisons included χ2 or Fisher’s tests, Student’s t-test, Mann-Whitney U test, and Kruskal-Wallis test. Interactions, confusion, and collinearity were thoroughly explored.

The impact of clinical and laboratory variables on 30-day all-cause mortality was first assessed using Cox regression analysis to confirm that SARS-CoV-2 RNAemia was independently associated with mortality in the whole cohort and the immunocompromised and immunocompetent sub-cohorts. Subsequently, the variables associated with the primary endpoint, SARS-CoV-2 RNAemia at COVID-19 diagnosis, and those considered clinically relevant were incorporated into a multiple logistic regression analysis. The variables in the models were selected manually using a backward stepwise process. Sensitivity analyses were performed, including changes in covariates and specific categorizations. The models of variables identified as associated with SARS-CoV-2 RNAemia in the multiple logistic regression analysis were evaluated using the area under the receiver-operating characteristic curve (AUC-ROC), its standard error (under the non-parametric assumption), and the asymptotic significance (being the null hypothesis a true area of 0.5). An AUC-ROC ≥ 0.70 was considered as evidence of good discrimination ability [21]. Missing data values (Supplementary S1 Table) were not imputed in our analysis given to their low number in the variables included in the multivariate analyses. Statistical analyses were performed using SPSS (version 26.0; IBM Corp., Armonk, New York, USA).

## Results

A whole cohort of 1011 patients with acute COVID-19 were included, consisting of 392 (38.8%) immunocompromised and 619 (61.2%) immunocompetent patient’s sub-cohorts (Table 1). In the Omicron period were included 377 (37.3%) patients, 78.1% of them immunocompromised, *vs.* in the Alpha and Delta periods in which were included 438 (43.3%) and 196 (19.4%) patients, 88.6% and 81.6% of them immunocompetent.

**Table 1:** Demographics, chronic underlying diseases, clinical characteristics and outcomes of COVID-19 in the entire cohort and in immunocompromised *vs.* immunocompetent patients.

Variables, N (%)	Whole cohort N = 1011	Immunocompromised N = 392	Immunocompetent N = 619	P
**Demographics and chronic underlying conditions**				
Male sex	629 (62.2)	234 (59.7)	395 (63.8)	0.188
Age (median, IQR)	64 (54-74)	65 (55-73)	64 (53-77)	0.549
Age > 70 years	354 (35.0)	134 (34.2)	220 (35.5)	0.659
Smoking (>10 packets/year)	101 (10.4)	61 (15.6)	40 (6.9)	**<0.001**
Diabetes mellitus	276 (27.3)	111 (28.3)	165 (26.7)	0.564
Chronic kidney disease	163 (16.2)	112 (28.6)	51 (8.3)	**<0.001**
Charlson Comorbidity Index ≥3	673 (66.6)	331 (84.4)	342 (55.3)	**<0.001**
Chronic prednisone therapy (>10 mg/day)	154 (18.2)	114 (31.1)	40 (8.3)	**<0.001**
***Variant of Concern (VOC) periods***				
Alpha	438 (43.3)	50 (11.4)^1^	388 (88.6)^1^	**<0.001**
Delta	196 (19.4)	36 (18.4)^1^	160 (81.6)^1^	
Omicron	377 (37.3)	306 (78.1)^1^	71 (11.5)^1^	
***COVID-19 vaccination in all patients and by VOC periods***				
All vaccinated patients	558 (55.2)	338 (86.2)	220 (35.5)	**<0.001**
Alpha	15 (2.7)	6 (40.0)^2^	9 (60.0)^2^	**<0.001**
Delta	181 (32.5)	34 (18.8)^2^	147 (81.2)^2^	
Omicron	362 (64.9)	298 (82.3)^2^	64 (17.7)^2^	
**Symptoms and signs at diagnosis**				
Fever	262 (26.5)	89 (29.4)	173 (25.3)	0.181
Cough	675 (66.9)	264 (67.5)	411 (66.5)	0.739
Dyspnea	484 (48.0)	163 (52.4)	321 (46.0)	0.059
SpO_2_ < 95%	377 (37.4)	130 (33.3)	247 (40.0)	**0.032**
Pneumonia	819 (82.0)	280 (71.4)	539 (88.8)	**<0.001**
CURB-65 ≥ 2	153 (23.8)	64 (30.8)	89 (20.4)	**0.004**
WHO clinical progression scale 6–9^3^	77 (7.6)	37 (9.4)	40 (6.5)	0.082
**Laboratory findings at diagnosis**				
Neutrophil count >7500/μL	195 (20.0)	69 (18.5)	126 (21.0)	0.350
Neutrophil count (median, IQR) (x1000)	4.54 (3.2-6.77)	4.23 (2.60-6.55)	4.74 (3.48-6.88)	**<0.001**
Lymphocyte count<1000/µL	546 (54.9)	260 (68.8)	286 (46.4)	**<0.001**
Lymphocyte count (median, IQR) (x1000)	0.93 (0.62-1.40)	0.71 (0.45-1.16)	1.04 (0.75-1.48)	**<0.001**
Platelets <130 000/μL	182 (18.3)	109 (28.8)	73 (11.8)	**<0.001**
Platelets (median, IQR)) (x1000)	189.0 (142.0-258.0)	166.5 (124.0-235.2)	206.0 (155.0-271.5)	**<0.001**
Creatinine >1.3 mg/dL	280 (28.2)	179 (47.4)	101 (16.4)	**<0.001**
Creatinine, mg/dL (median [IQR])	0.95 (0.74-1.41)	1.27 (0.87-1.93)	0.88 (0.70-1.11)	**<0.001**
C-reactive protein > 100 mg/L	344 (35.0)	140 (37.4)	204 (33.4)	0.203
C-reactive protein mg/L (median [IQR])	66.2 (27.1-132.0)	75.5 (32.7-135.1)	63.4 (25.4-129.2)	**0.018**
D-dimer >600 ng/mL	463 (55.2)	199 (67.5)	264 (48.5)	**<0.001**
D-dimer (median, IQR)	670 (380-1244)	860 (520-1650)	570 (330-1077.5)	**<0.001**
LDH > 300 IU/L	446 (47.0)	169 (48.7)	277 (46.0)	0.424
LDH IU/L (median [IQR])	291 (232-368)	295 (227-377)	288.5 (236-365)	0.739
RNAemia	311 (30.8)	195 (49.7)	116 (18.7)	**<0.001**
IgM positive	338 (54.1)	80 (36.9)	258 (63.2)	**<0.001**
IgM ng/ml (median, IQR)	92.4 (57.5-222.2)	77.4 (42.6-180.7)	94.5 (60.0-242.3)	**0.039**
IgG positive	531 (85.1)	176 (82.2)	355 (86.6)	0.148
IgG ng/ml (median, IQR)	2088.8 (612.9-3592.9)	1531.9 (348.8-3180.9)	2279.0 (750.7-3621.3)	**0.014**
Neutralising antibodies absence	232 (35.7)	180 (50.7)	52 (17.7)	**<0.001**
Neutralising antibodies (GMT, IC95)	216.1 (175.3-267.1)	107.0 (77.34-147.6)	359.4 (272.5-472.9)	**<0.001**
IFN-α undetectable	86 (11.5)	32 (9.4)	54 (13.3)	0.092
IFN-α pg/mL (median, IQR)	23.75 (11.76-60.62)	43.70 (19.59-81.50)	18.32 (9.28-29.43)	**<0.001**
IFN-γ undetectable	215 (25.2)	124 (35.0)	91 (18.3)	**<0.001**
IFN-γ pg/mL (median, IQR)	69.12 (17.27-182.09)	52.74 (9.84-84.90)	106.97 (24.96-272.88)	**<0.001**
**Treatment**				
Antiviral	269 (27.0)	179 (47.5)	90 (14.6)	**<0.001**
Remdesivir	208 (20.9)	118 (31.3)	90 (14.6)	**<0.001**
Tocilizumab	123 (12.4)	56 (14.9)	67 (10.8)	0.062
Dexamethasone	558 (62.1)	216 (57.3)	342 (65.6)	**0.011**
Antibiotics	180 (20.0)	97 (27.2)	83 (15.3)	**<0.001**
**Outcome**				
Hospital admission	969 (95.8)	358 (91.3)	611 (98.7)	**<0.001**
Length of hospital stay (days, median [IQR])	7 (4-12)	8 (5-14.5)	6 (4-10)	**<0.001**
HFNO^4^	48 (4.8)	24 (6.1)	24 (3.9)	0.106
Intensive care unit admission	76 (7.5)	32 (8.2)	44 (7.1)	0.505
WHO clinical progression scale 7–10^3^	129 (12.8)	58 (14.8)	71 (11.5)	0.123
Mortality at day + 30	79 (7.8)	47 (12.0)	32 (5.2)	**<0.001**

^1^Percentages respect to the patients in each VOC period; ^2^ Percentages respect to the vaccinated patients in each VOC period; ^3^ https://doi.org/10.1016/S1473-3099(20)30483-7; ^4^ HFNO: High-Flow Nasal Oxygen.

Regarding the characteristics of COVID-19 at diagnosis in the whole cohort, 96.6% patients were hospital-admitted, 82.0% had pneumonia, 7.9% a WHO clinical progression scale score of 6–9, 30.8% SARS-CoV-2 RNAemia, and the 30-day all-cause mortality was 7.8%. Comparing both sub-cohorts, immunocompromised had been vaccinated against SARS-CoV-2 more frequently than immunocompetent patients (86.2% and 35.5%, p < 0.001). SARS-CoV-2 RNAemia was more frequent in immunocompromised than in immunocompetent (49.7% and 18.7%, p < 0.001) sub-cohorts, as well as immunocompromised patients had higher 30-day all-cause mortality (12.0% and 5.2%, p < 0.001).

### Variables associated with 30-day all-cause mortality in the whole cohort and immunocompromised and immunocompetent sub-cohorts

The variables associated with 30-day all-cause mortality in the univariate analysis, in the whole cohort and immunocompromised and immunocompetent sub-cohorts, are detailed in Supplementary S2 Tables, S3, and S4. In dead patients, SARS-CoV-2 RNAemia was more frequent in the whole cohort (65.8% vs. 27.8%; p < 0.001) and the immunocompromised (80.9% vs. 45.5%; p = 0.004) and immunocompetent (43.8% vs. 17.4%, respectively; p = 0.051) sub-cohorts. Regarding the SARS-CoV-2 VOC, mortality was lower in immunocompromised patients included in the Omicron period (7.8%) than in Alpha (28%) and Delta (25.0%) periods (p < 0.001), without differences in the immunocompetent patients.

Subsequently, different Cox multiple regression models were generated to identify independent factors associated with 30-day all-cause mortality for the whole cohort and both sub-cohorts (Table 2). For the whole cohort, male sex (hazard ratio [HR]: 0.48 [95% CI 0.30–0.75]), Charlson comorbidity index ≥ 3 (HR: 5.43 [2.19–13.46]), dyspnea (HR: 1.81 [1.13–2.92]), CRP > 100 mg/L (HR: 1.70 [1.08–2.67]) and RNAemia (HR: 2.19 [1.37–3.51]) were selected in the final model. In the immunocompromised sub-cohort, independent variables associated with mortality were male sex (HR: 0.37 [0.20–0.68]), age > 70 years (HR: 2.98 [1.65–5.38]), Alpha and Delta VOVC periods (HR: 3.10 [1.74–5.53]), dyspnea (HR: 1.97 [1.06–3.64]) and RNAemia (HR: 3.30 [1.57–6.93]). Finally, in the immunocompetent sub-cohort selected variables were Charlson comorbidity index ≥ 3 (HR: 22.42 [3.06–164.39]) and RNAemia (HR: 2.35 (1.16–4.75), p = 0.018). The administration of remdesivir, tocilizumab, and dexamethasone were not included in the multivariate Cox regression analysis because of they were prescribed, at the criteria of the attending physicians, more frequently in case with severe disease at diagnosis (Supplementary S5 Table) in the whole cohort and both sub-cohorts.

**Table 2:** Variables associated with 30-day all-cause mortality in the whole cohort and in the immunocompromised and immunocompetent sub-cohorts: Multivariate Cox regression analyses.

Whole cohort		
***Variables, deaths = 79***	**HR (95% CI)**	**P**
Male sex	0.48 (0.30-0.75)	**0.001**
Charlson Comorbidity Index ≥3	5.43 (2.19-13.46)	**<0.001**
Dyspnea	1.81 (1.13-2.92)	**0.014**
C-reactive protein > 100 mg/L	1.70 (1.08-2.67)	**0.021**
RNAemia	2.19 (1.37-3.51)	**0.001**
**Immunocompromised sub-cohort**		
***Variables, deaths = 47***	**HR (95% CI)**	**P**
Male sex	0.37 (0.20-0.68)	**0.002**
Age > 70 years	2.98 (1.65-5.38)	**<0.001**
Alpha and Delta VOC periods	3.10 (1.74-5.53)	**<0.001**
Dyspnea	1.97 (1.06-3.64)	**0.031**
RNAemia	3.30 (1.57-6.93)	**0.002**
**Immunocompetent sub-cohort**		
***Variables, deaths = 32***	**HR (95% CI)**	**P**
Charlson Comorbidity Index ≥3	22.42 (3.06-164.39)	**0.002**
RNAemia	2.35 (1.16-4.75)	**0.018**

### Variables associated with SARS-CoV-2 RNAemia in the whole cohort and in immunocompromised and immunocompetent sub-cohorts

Variables associated with SARS-CoV-2 RNAemia in the whole cohort and immunocompromised and immunocompetent sub-cohorts are detailed in Table 3 and Supplementary S6 Tables and S8. In the whole cohort (Table 3), patients with SARS-CoV-2 RNAemia had higher frequencies of immunocompromise (p < 0.001) and RNAemia was more frequent in the Omicron than Alpha or Delta VOC periods (p < 0.001). Multiple logistic regression analysis of the whole cohort (Table 4) showed that immunocompromise (odds ratio [OR]: 3.66 [2.47–5.44]), lymphocyte count < 1000/µL (OR: 1.60 [1.16–2.19]) and LDH > 300 UI/L (OR: 2.51 [1.84–3.42]) were independently associated with the presence of SARS-CoV-2 RNAemia at COVID-19 diagnosis. RNAemia seems independently associated with the Omicron VOC period, but this effect was no longer present (OR: 1.44 [0.97–2.15]) when SpO_2_ was removed from the model. The AUC-ROC were 0.763 (0.731−0795, p < 0.001) and 0.759 (0.726–0.791, p < 0.001) for both models (Supplementary S1 Fig).

**Table 3:** Demographics, chronic underlying diseases, clinical characteristics and outcomes of patients in the whole cohort with *vs.* without SARS-CoV-2 RNAemia at COVID-19 diagnosis.

Variables	Whole cohort N = 1011	RNAemia N = 311 (30.8%)	No RNAemia N = 700 (69.2%)	P
**Demographics and chronic underlying conditions**				
Male sex	629 (62.2)	200 (64.3)	429 (61.3)	0.360
Age (median, IQR)	64 (54-74)	66 (57-74)	63 (52-75)	0.081
Age > 70 years	354 (35.0)	111 (35.7)	243 (34.7)	0.764
Smoking (>10 packets/year)	101 (10.4)	38 (12.3)	63 (9.5)	0.178
Diabetes mellitus	276 (27.3)	93 (29.9)	183 (26.1)	0.215
Chronic kidney disease	163 (16.2)	73 (23.6)	90 (12.9)	**<0.001**
Charlson Comorbidity Index ≥3	673 (66.6)	237 (76.2)	436 (62.3)	**<0.001**
Chronic prednisone therapy (>10 mg/day)	154 (18.2)	71 (25.3)	83 (14.7)	**<0.001**
Immunocompromise	392 (38.8)	195 (62.7)	197 (28.1)	**<0.001**
***Variant of Concern (VOC) periods***				
Alpha	438 (43.3)	87 (19.9)^1^	351 (80.1)^1^	**<0.001**
Delta	196 (19.4)	52 (26.5)^1^	144 (73.5)^1^	
Omicron	377 (37.3)	172 (45.6)^1^	205 (54.4)^1^	
***COVID-19 vaccination in all patients and by VOC periods***				
All vaccinated patients	558 (55.2)	220 (70.7)	338 (48.3)	**<0.001**
Alpha	15 (2.7)	5 (33.3)^2^	10 (66.7)^2^	**<0.001**
Delta	181 (32.4)	49 (27.1)^2^	132 (72.9)^2^	
Omicron	362 (64.9)	166 (45.9)^2^	196 (54.1)^2^	
**Symptoms and signs at diagnosis**				
Fever	262 (26.5)	89 (29.4)	173 (25.3)	0.181
Cough	675 (66.9)	224 (72.3)	451 (64.5)	**0.016**
Dyspnoea	484 (48.0)	163 (52.4)	321 (46.0)	0.059
SpO_2_ < 95%	377 (37.4)	142 (45.7)	235 (33.8)	**<0.001**
Pneumonia	819 (82.0)	258 (84.0)	561 (81.1)	0.260
CURB-65 ≥ 2	153 (23.8)	64 (30.8)	89 (20.4)	**0.004**
WHO clinical progression scale 6–9^3^	77 (7.6)	46 (14.8)	31 (4.4)	**<0.001**
**Laboratory findings at diagnosis**				
Neutrophil count >7500/μL	195 (20.0)	66 (21.9)	129 (19.2)	0.320
Neutrophil count (median, IQR) (x1000)	4.5 (3.2-6.8)	4.3 (2.9-7.1)	4.6 (3.3-6.6)	0.265
Lymphocyte count<1000/µL	546 (54.9)	211 (68.5)	335 (48.8)	**<0.001**
Lymphocyte count (median, IQR) (x1000)	0.9 (0.6-1.4)	0.7 (0.5-1.2)	1.0 (0.7-1.5)	**<0.001**
Platelets <130 000/μL	182 (18.3)	75 (24.4)	107 (15.6)	**0.001**
Platelets (median, IQR)) (x1000)	189.0 (142.0-258.0)	172.5 (131.0-236.7)	201.0 (148.0-268.0)	**<0.001**
Creatinine >1.3 mg/dL	280 (28.2)	126 (41.2)	154 (22.4)	**<0.001**
Creatinine, mg/dL (median [IQR])	0.9 (0.7-1.4)	1.1 (0.8-1.7)	0.9 (0.7-1.2)	**<0.001**
C-reactive protein > 100 mg/L	344 (35.0)	145 (47.7)	199 (29.3)	**<0.001**
C-reactive protein mg/L (median [IQR])	66.2 (27.1-132.0)	94.1 (47.1-158.3)	56.8 (22.2-112.0)	**<0.001**
D-dimer >600 ng/mL	463 (55.2)	157 (63.3)	306 (51.8)	**0.002**
D-dimer (median, IQR)	670.0 (380.0-1244.0)	750.0 (472.5-1355.0)	640.0 (340.0-1150.0)	**<0.001**
LDH > 300 IU/L	446 (47.0)	182 (62.1)	264 (40.2)	**<0.001**
LDH IU/L (median [IQR])	291.0 (232.0-368.0)	328.0 (257.0-439.5)	276.0 (227.0-344.0)	**<0.001**
IgM positive	338 (54.1)	85 (42.9)	253 (59.3)	**<0.001**
IgM ng/ml (median, IQR)	92.4 (57.5-222.2)	86.2 (53.2-181.4)	94.1 (59.5-223.4)	0.364
IgG positive	531 (85.1)	164 (83.2)	367 (85.9)	0.379
IgG ng/ml (median, IQR)	2088.8 (612.9-3592.9)	1748.0 (490.3-3423.7)	2128.9 (743.2-3688.2)	0.093
Neutralising antibodies absence	232 (35.7)	128 (51.0)	104 (26.1)	**<0.001**
Neutralising antibodies (GMT, IC95)	216.1 (174.2-264.9)	140.7 (99.11-198.0)	258.7 (201.4-337.3)	**0.016**
IFN-α undetectable	86 (11.5)	24 (9.8)	62 (12.4)	0.296
IFN-α pg/mL (median, IQR)	23.75 (11.76-60.62)	38.91 (17.44-77.06)	20.84 (10.85-45.74)	**<0.001**
IFN-γ undetectable	215 (25.2)	80 (29.0)	135 (23.4)	0.081
IFN-γ pg/mL (median, IQR)	69.12 (17.27-182.09)	60.29 (12.80-147.21)	72.5 (22.12-226.21)	**0.007**
**Treatment**				
Antiviral	269 (27.0)	113 (37.7)	156 (22.4)	**<0.001**
Remdesivir	208 (20.9)	90 (30.0)	118 (17.0)	**<0.001**
Tocilizumab	123 (12.4)	77 (25.7)	46 (6.6)	**<0.001**
Dexamethasone	558 (62.1)	214 (74.0)	344 (56.5)	**<0.001**
Antibiotics	180 (20.0)	77 (27.9)	103 (16.5)	**<0.001**
**Outcome**				
Hospital admission	969 (95.8)	299 (96.1)	670 (95.7)	0.753
Length of hospital stay (days, median [IQR])	7 (4-12)	10 (5-15)	6 (4-10)	**<0.001**
HFNO^4^	48 (4.8)	31 (10.0)	17 (2.4)	**<0.001**
Intensive care unit admission	76 (7.5)	42 (13.6)	34 (4.9)	**<0.001**
WHO clinical progression scale 7–10^3^	129 (12.8)	75 (24.1)	54 (7.7)	**<0.001**
Mortality at day + 30	79 (7.8)	52 (16.7)	27 (3.9)	**<0.001**

^1^Percentages respect to the patients in each VOC period; ^2^ Percentages respect to the vaccinated patients in each VOC period; ^3^ https://doi.org/10.1016/S1473-3099(20)30483-7; ^4^ HFNO: High-Flow Nasal Oxygen.

**Table 4:** Risk factors associated with the presence of RNAemia in the whole cohort and in the immunocompromised and immunocompetent sub-cohorts: Multivariate logistic regression analyses.

*Whole cohort*		
***Model A, N = 945***	**OR (95% CI)**	**P**
Immunocompromise	3.74 (2.51-5.58)	**<0.001**
Omicron period	1.55 (1.03-2.32)	**0.035**
SpO_2_ < 95%	1.67 (1.21-2.30)	**0.002**
Lymphocyte count<1000/µL	1.54 (1.12-2.11)	**0.008**
LDH > 300 IU/L	2.25 (1.64-3.09)	**<0.001**
***Model B, N = 948***	**OR (95% CI)**	**P**
Immunocompromise	3.66 (2.47-5.44)	**<0.001**
Omicron VOC period	1.44 (0.97-2.15)	0.071
Lymphocyte count<1000/µL	1.60 (1.16-2.19)	**0.004**
LDH > 300 IU/L	2.51 (1.84-3.42)	**<0.001**
**Immunocompromised sub-cohort**		
***N = 317***	**OR (95% CI)**	**P**
Alpha and Omicron VOC periods	1.95 (1.01-3.79)	**0.048**
Pneumonia	1.96 (1.10-3.50)	**0.023**
LDH > 300 IU/L	1.64 (1.02-2.63)	**0.041**
Neutralizing antibodies absence	2.51 (1.57-4.00)	**<0.001**
**Immunocompetent sub-cohort**		
***N = 605***	**OR (95% CI)**	**P**
Delta and Omicron VOC periods	2.27 (1.46-3.52)	**<0.001**
Lymphocyte count<1000/µL	1.81 (1.16-2.80)	**0.008**
LDH > 300 IU/L	3.99 (2.51-6.36)	**<0.001**

In the immunocompromised sub-cohort (Supplementary S6 Table), SARS-CoV-2 RNAemia was more frequent in the Alpha or Omicron than in Delta VOC periods (p = 0.05). Multiple logistic regression analysis (Table 4) identified the Alpha and Omicron VOC periods (OR: 1.95 [1.01–3.79]), pneumonia (OR: 1.96 [1.10–3.50]), LDH > 300 UI/L (OR: 1.64 [1.02–2.63]) and the absence of neutralizing antibodies (OR: 2.51 [1.57–4.00]) as independent factors associated with the presence of SARS-CoV-2 RNAemia at COVID-19 diagnosis, with an AUC-ROC of 0.678 (0.619–0.737, p < 0.001) (Supplementary S1 Fig).

The immunocompromised sub-cohort includes different types of immunocompromise (Supplementary S3 Table) without difference in mortality rates (p = 0.429). However, RNAemia was more frequent (p = 0.045) in hematological malignancies (HM) and solid organ transplant recipients (SOT) than in solid neoplasia (SN) and other causes of immunocompromise (Supplementary S6 Table). As a sensitivity analysis, we next analyzed factors associated with the presence of RNAemia in these different immunocompromised patients (Supplementary S7 Table). Pneumonia (OR: 2.53 [1.18–5.40]) in HM, pneumonia (OR: 2.50 [1.15–5.47]) and neutralizing antibodies absence (OR: 2.81 [1.48–5.35]) in SOT, and neutralizing antibodies absence (OR: 5.37 [1.32–21.87]) in SN were factors independently associated with RNAemia.

SARS-CoV-2 RNAemia in the immunocompetent sub-cohort (Supplementary S8 Table) was more frequent in the Delta and Omicron than in Alpha VOC periods (p = 0.003). Factors independently associated with RNAemia were Delta and Omicron VOC periods (OR: 2.27 [1.46–3.52]), lymphocyte count < 1000/µL (OR: 1.81 [1.16–2.80]) and LDH levels > 300 IU/L (OR: 3.99 [2.51–6.36]) (Table 4). The model has an AUC-ROC of 0.720 (0.670–0.770, p < 0.001) (Supplementary S1 Fig).

## Discussion

The present study identifies factors associated with the SARS-CoV-2 RNAemia at the COVID-19 diagnosis. Regarding the host underlying condition, diseases causing immunocompromise are the most prominent ones in the whole cohort. Among the virologic factors, patients included in the Omicron VOC period have more RNAemia, both in immunocompromised and immunocompetent patients. Pneumonia in immunocompromised patients, high serum LDH levels in all patients, and markers of lower immune response, neutralizing antibodies absence in immunocompromised and lymphopenia in immunocompetent patients, were also associated with RNAemia.

In the present study, as a first analysis, we confirmed that SARS-CoV-2 RNAemia was an independent risk factor for 30-day all-cause mortality in both immunocompromised and immunocompetent individuals. Our group and others have previously evaluated the relevance of RNAemia in monitoring COVID-19 patients. The rate of SARS-CoV-2 RNAemia is highly variable in the literature, ranging from 10% to over 60% [3–9,18,22–30], which could be explained by the differences found among the demographic characteristics, underlying conditions, and time from symptom onset. The rate of RNAemia in this study was in line with previous reports by our group and colleagues in both immunocompetent [9,22,23] and immunocompromised patients [9,18]. In addition, despite most patients being fully vaccinated since June 2021, especially in the immunocompromised group, the incidence of RNAemia remained elevated at close to four out of ten cases. The mortality rate in our study was lower than that in previous studies conducted by our group in non-vaccinated immunocompromised and immunocompetent patients [9,22], which is in line with other studies in pre-vaccination cohorts [6,31]. As previously observed in a cohort of 408 immunocompetent individuals and 47 solid organ transplant recipients [9], RNAemia was independently associated with mortality in all immunocompetent or immunocompromised patients.

The increased frequency of RNAemia and its strong association with mortality in immunocompromised patients may reflect impaired viral control and persistent systemic inflammation, which together could facilitate viral dissemination. Weak immune responses may not efficiently eliminate viral replication or bloodstream dissemination. Another associated factor is LDH, an enzyme involved in inflammatory processes, which in turn leads to tissue damage. This could explain the bloodstream release and dissemination of the virus [32,33]. Moreover, higher LDH levels have been associated with severity of COVID-19 pneumonia [34]. In addition, in immunocompetent patients, lymphopenia was independently associated with RNAemia, which is explained by the implication of lymphocytes in the innate immune response, including lymphoid cells type 1 as the first line of defense against viruses. In a previous Spanish nationwide study in a cohort of 4035 patients with COVID-19, lymphopenia was more common in patients who died, being an important predictor of mortality [31] similar to RNAemia, which was also associated with lymphopenia in other studies [4,5,9].

In immunocompromised patients, despite vaccination, the absence of neutralizing antibodies was independently associated with RNAemia. The absence of neutralizing antibodies seems to favor viral dissemination and, in turn, increase tissue damage, which explain the leakage of viral particles and their components from the tissues into the bloodstream [35]. This finding was observed in immunocompromised patients as a whole and, specifically, in both SOT recipients and patients with solid neoplasia. In patients with hematological malignancies, with a vaccination rate similar to the other immunocompromised patients, RNAemia was associated with the presence of pneumonia, as previously reported [18], but not with the absence of neutralizing antibodies; this fact may be explained by the heterogeneity of diseases included in this type on immunocompromised patients.

Our study has several limitations. Although it was a prospective cohort, inclusion was dependent on having blood samples to evaluate RNAemia after obtaining informed consent from patients. Additionally, the number of patients with mild disease not requiring hospital admission was smaller than the number of patients admitted to the hospital, although more patients had moderate rather than severe disease at the time of blood sampling. Another limitation was the different vaccination rates between immunocompromised patients, who were the first scheduled to receive the vaccine, and immunocompetent patients. Despite these limitations, the strength of this study lies in the multicenter design of our cohort, which was recruited from six Spanish hospitals. Each hospital had its own clinical practice and protocols, although the clinical practice was similar because all followed common national and international guidelines, confirmed by comparing the treatments received by patients with severe and mild patients. Immunocompromised and immunocompetent patients with clinically severe COVID-19, which has previously been associated with RNAemia [4,6,9], were treated more frequently with tocilizumab and dexamethasone at the discretion of the physicians in charge. Finally, the present study has not considered the possible viral and bacterial co-infections [36]. However, the accuracy of co-infections diagnosis in the viral community-acquired respiratory infections, including pneumonia, is a difficult issue because of the lack of specificity of many bacterial isolates from respiratory clinical samples. A study on 50,419 upper respiratory samples, positive for SARS-CoV-2, detected 4% and 33% of viral and bacterial co-infections, respectively, but without expressing the method to differentiate *Staphylococcus aureus* or *Haemophilus influenzae* detection, as examples, from asymptomatic carriers [37]. In addition, a review of 13 studies disclosed a wide co-infection and secondary infection rates in SARS-CoV-2 infection, from 0.6% to 45.0% [38], which denotes the very different criteria for diagnostic specificity used in the different studies.

## Conclusions

The results of this study show that the main factor determining the presence of SARS-CoV-2 RNAemia at COVID-19 diagnosis depends on underlying conditions such as immunodeficiency, with RNAemia rates almost three times higher in this population than those in immunocompetent individuals. Pneumonia and absence of neutralizing antibodies in immunocompromised patients, biomarkers of tissue damage as LDH in all patients, and lymphopenia in immunocompetent patients were also associated with bloodstream dissemination of the virus. These data highlight the need for quick detection of SARS-CoV-2 RNAemia for early initiation of antiviral treatment, especially in the most vulnerable populations, as RNAemia is associated with poor clinical outcomes. The efficacy of antiviral treatments, through clinical studies, must consider the different demographics features and chronic underlying diseases of patients.

## Supporting information

S1 Additional supporting information may be found online in the Supporting Information section. (DOCX)
