# Supplementary material for: Factors associated with SARS-CoV-2 RNAemia development at COVID-19 diagnosis
Source: PLoS One. 2025 Aug 21;20(8):e0330495. doi: 10.1371/journal.pone.0330495 (PMC12370080; doi:10.1371/journal.pone.0330495)

**Factors associated with SARS-CoV-2 RNAemia development at COVID-19 diagnosis**

**Supplementary material**

**Table S1 a S8**

**Figure S1**

**Table S1**. Missing values data for the variables collected in the cohort.

| **Variables** | **Missing data (%)** | | |
| --- | --- | --- | --- |
|  | **Whole cohort** | **Immunocompromised** | **Immunocompetent** |
| **Demographics and chronic underlying conditions** | | | |
| Male sex | 0 | 0 | 0 |
| Age | 0 | 0 | 0 |
| Smoking (>10 packets/year) | 3.96 | 0.25 | 6.3 |
| Diabetes mellitus | 0 | 0 | 0 |
| Chronic kidney disease | 0.20 | 0.25 | 0.16 |
| Charlson Comorbidity Index ≥3 | 0 | 0 | 0 |
| Chronic prednisone therapy (>10 mg/day) | 16.22 | 6.37 | 22.45 |
| Immunocompromise | 0 | 0 | 0 |
| Type of immunocompromise | 0.76 | 0.77 | - |
| COVID-19 vaccination | 0 | 0 | 0 |
| **Symptoms and signs at diagnosis** | | | |
| Fever | 2.37 | 6.12 | 0 |
| Cough | 0.20 | 0.25 | 0.16 |
| Dyspnea | 0.20 | 0 | 0.32 |
| SpO2 <95% | 0.40 | 0.5 | 0.32 |
| Pneumonia | 1.19 | 0 | 1.93 |
| CURB-65 ≥2 (in patients with pneumonia) | 21.37 | 20.71 | 21.7 |
| WHO clinical progression scale 6-9 ^1^ | 0 | 0 | 0 |
| **Laboratory findings at diagnosis** | | | |
| Neutrophils count | 3.66 | 4.85 | 2.91 |
| Lymphocytes count | 1.58 | 3.57 | 0.32 |
| Platelets count | 1.58 | 3.57 | 0.32 |
| Creatinine | 1.88 | 3.57 | 0.81 |
| C-reactive protein | 2.67 | 4.59 | 1.45 |
| D-dimer | 17.01 | 24.75 | 12.11 |
| LDH | 6.13 | 11.47 | 2.74 |
| RNAemia | 0 | 0 | 0 |
| IgM | 38.28 | 44.64 | 34.09 |
| IgG | 38.28 | 45.4 | 33.76 |
| Neutralizing antibodies | 35.81 | 9.43 | 52.5 |
| IFN-α | 26.01 | 12.76 | 34.4 |
| IFN-γ | 15.73 | 9.69 | 19.55 |
| **Treatments** | | | |
| Antivirals | 1.58 | 3.82 | 0.16 |
| Remdesivir | 1.58 | 3.82 | 0.16 |
| Tocilizumab | 1.58 | 3.82 | 0.16 |
| Dexamethasone | 11.17 | 3.82 | 15.83 |
| Antibiotics | 11.08 | 9.18 | 12.28 |
| **Outcomes** | | | |
| Hospital admission | 0 | 0 | 0 |
| Length of hospital stay | 4.25 | 8.67 | 1.29 |
| HFNO | 0.30 | 0 | 0.48 |
| Intensive care unit admission | 0.40 | 1.02 | 0 |
| WHO clinical progression scale 7–10 ^1^ | 0 | 0 | 0 |
| Mortality at day +30 | 0 | 0 | 0 |
| Abbreviations (in order of appearance): SpO2, peripheral capillary oxygen saturation; CURB-65, Severity Score for Community-Acquired Pneumonia; LDH, lactate dehydrogenase; Ig, Immunoglobulin; IFN, interferon; HFNO: High-Flow Nasal Oxygen.  ^1^ WHO Clinical Progression Scale, <https://doi.org/10.1016/S1473-3099(20)30483-7> | | | |

**Table S2.** Demographics, chronic underlying diseases, clinical characteristics and outcomes of the whole cohort according to 30-day all-cause mortality.

| **Variables** | **Whole cohort**  **N = 1011** | **Dead**  **N = 79 (7.8%)** | **Survivors**  **N = 932 (92.2%)** | **P** |
| --- | --- | --- | --- | --- |
| **Demographics and chronic underlying conditions** | | | | |
| Male sex | 629 (62.2) | 39 (49.4) | 590 (63.3) | **0.002** |
| Age (median, IQR) | 64 (54-74) | 74 (66-80) | 63 (53-74) | **<0.001** |
| Age >70 years | 354 (35.0) | 49 (62.0) | 305 (32.7) | **<0.001** |
| Smoking (>10 packets/year) | 101 (10.4) | 10 (12.7) | 91 (10.2) | 0.643 |
| Diabetes mellitus | 276 (27.3) | 29 (36.7) | 247 (26.5) | 0.106 |
| Chronic kidney disease | 163 (16.2) | 22 (27.8) | 141 (15.2) | **0.046** |
| Charlson Comorbidity Index ≥3 | 673 (66.6) | 74 (93.7) | 599 (64.3) | **<0.001** |
| Chronic prednisone therapy (>10 mg/day) | 154 (18.2) | 18 (27.7) | 136 (17.4) | 0.432 |
| Immunocompromise | 392 (38.8) | 47 (59.5) | 345 (37.0) | 0.078 |
| ***Variant of Concern periods*** | | | | |
| Alpha | 438 (43.3) | 31 (7.1) ^1^ | 407 (92.9) ^1^ | 0.241 |
| Delta | 196 (19.4) | 21 (10.7) ^1^ | 175 (89.3) ^1^ |  |
| Omicron | 377 (37.3) | 27 (7.2) ^1^ | 350 (92.8) ^1^ |  |
| ***COVID-19 vaccination in all patients and by VOC periods*** | | | | |
| All vaccinated patients | 558 (55.2) | 49 (62.0) | 509 (54.6) | 0.203 |
| Alpha | 15 (2.7) | 3 (20.0) ^2^ | 12 (80.0) ^2^ | 0.097 |
| Delta | 181 (32.5) | 20 (11.0) ^2^ | 161 (90.0) ^2^ |  |
| Omicron | 362 (64.9) | 26 (7.2) ^2^ | 336 (92.8) ^2^ |  |
| **Symptoms and signs at diagnosis** | | | | |
| Fever | 262 (26.5) | 21 (26.6) | 241 (26.5) | 0.793 |
| Cough | 675 (66.9) | 53 (67.1) | 622 (66.9) | 0.472 |
| Dyspnea | 484 (48.0) | 54 (68.4) | 430 (46.2) | **0.007** |
| SpO_2_ <95% | 377 (37.4) | 41 (51.9) | 336 (36.2) | 0.598 |
| Pneumonia | 819 (82.0) | 70 (88.6) | 749 (81.4) | 0.973 |
| CURB-65 ≥2 | 153 (23.8) | 34 (59.6) | 119 (20.3) | **<0.001** |
| WHO clinical progression scale 6-9 ^3^ | 77 (7.6) | 19 (24.1) | 58 (6.2) | 0.106 |
| **Laboratory findings at diagnosis** | | | | |
| Neutrophil count >7500/μL | 195 (20.0) | 19 (24.7) | 176 (19.6) | 0.629 |
| Neutrophil count (median, IQR) (x1000) | 4.5 (3.2-6.8) | 5.2 (3.2-7.4) | 4.5 (3.2-6.7) | 0.567 |
| Lymphocyte count<1000/µL | 546 (54.9) | 58 (73.4) | 488 (53.3) | 0.168 |
| Lymphocyte count (median, IQR) (x1000) | 0.9 (0.6-1.4) | 0.6 (0.5-1.1) | 1.0 (0.6-1.4) | 0.653 |
| Platelets <130 000/μL | 182 (18.3) | 14 (17.7) | 168 (18.3) | 0.236 |
| Platelets  (median, IQR) ) (x1000) | 189.0  (142.0-258.0) | 167.0  (137.0-215.0) | 194.5  (144.0-260.0) | 0.707 |
| Creatinine >1.3 mg/dL | 280 (28.2) | 39 (49.4) | 241 (26.4) | **0.028** |
| Creatinine, mg/dL (median [IQR]) | 0.9 (0.7-1.4) | 1.3 (1.0-2.1) | 0.9 (0.7-1.3) | 0.059 |
| C-reactive protein > 100 mg/L | 344 (35.0) | 42 (53.2) | 302 (33.4) | **0.032** |
| C-reactive protein mg/L  (median [IQR]) | 66.2  (27.1-132.0) | 111.3  (60.0-200.0) | 64.2  (25.5-126.4) | **0.001** |
| D-dimer >600 ng/mL | 463 (55.2) | 45 (70.3) | 418 (53.9) | 0.277 |
| D-dimer  (median, IQR) | 670.0  (380.0-1244.0) | 996.0  (532.5-1765.0) | 660.0  (370.0-1160.0) | 0.837 |
| LDH >300 IU/L | 446 (47.0) | 48 (62.3) | 398 (45.6) | 0.267 |
| LDH IU/L (median [IQR]) | 291.0  (232.0-368.0) | 342.0  (271.5-448.5) | 287.0  (231.0-363.7) | 0.666 |
| IgM positive | 338 (54.1) | 25 (54.3) | 313 (54.1) | 0.180 |
| IgM ng/ml  (median, IQR) | 92.4  (57.5-222.2) | 86.2  (71.5-170.2) | 93.8  (56.9-223.4) | 0.660 |
| IgG positive | 531 (85.1) | 38 (80.9) | 493 (85.4) | 0.769 |
| IgG ng/ml  (median, IQR) | 2088.8  (612.9-3592.9) | 1936.6  (316.7-3506.6) | 2093.0  (635.4-3612.8) | 0.756 |
| Neutralizing antibodies absence | 232 (35.7) | 37 (56.9) | 195 (33.4) | 0.110 |
| Neutralizing antibodies (GMT, IC95) | 216.1  (174.2-264.9) | 118.4  (58.0-253.5) | 225.7  (179.1-280.5) | 0.288 |
| IFN-α undetectable | 86 (11.5) | 6 (9.7) | 80 (11.7) | 0.862 |
| IFN-α pg/mL (median, IQR) | 23.7  (11.7-60.6) | 29.7 (19.8-74.7) | 23.5 (11.5-58.3) | **<0.001** |
| IFN-γ undetectable | 215 (25.2) | 24 (35.8) | 191 (24.3) | 0.153 |
| IFN-γ pg/mL (median, IQR) | 69.1  (17.3-182.1) | 82.4  (37.8-252.8) | 67.1  (17.0-180.7) | **0.036** |
| RNAemia | 311 (30.8) | 52 (65.8) | 259 (27.8) | **<0.001** |
| **Treatment** | | | | |
| Antiviral | 269 (27.0) | 16 (22.9) | 253 (27.4) | 0.064 |
| Remdesivir | 208 (20.9) | 15 (21.4) | 193 (20.9) | 0.114 |
| Tocilizumab | 123 (12.4) | 25 (35.7) | 98 (10.6) | **0.021** |
| Dexamethasone | 558 (62.1) | 51 (75.0) | 507 (61.1) | 0.377 |
| Antibiotics | 180 (20.0) | 21 (35.0) | 159 (19.0) | 0.217 |
| **Outcome** | | | | |
| Hospital admission | 969 (95.8) | 79 (100) | 890 (95.3) | - |
| Length of hospital stay  (days, median [IQR] | 7.0 (4.0-12.0) | 14.0 (6.0-19.0) | 7.0 (4.0-11.0) | **<0.001** |
| HFNO ^4^ | 48 (4.8) | 19 (24.1) | 29 (3.1) | 0.121 |
| Intensive care unit admission | 76 (7.5) | 17 (22.7) | 59 (6.3) | 0.395 |
| WHO clinical progression scale 7–10 ^3^ | 129 (12.8) | 7.9 (100) | 50 (5.4) | **<0.001** |

^1^ Percentages respect to the patients in each VOC period; ^2^ Percentages respect to the vaccinated patients in each VOC period; ^3^ <https://doi.org/10.1016/S1473-3099(20)30483-7>; ^4^ HFNO: High-Flow Nasal Oxygen.

**Table S3.** Demographics, chronic underlying diseases, clinical characteristics and outcomes of immunocompromised sub-cohort according to 30-day all-cause mortality.

| **Variables** | **Immunocompromised sub-cohort**  **N = 392** | **Dead**  **N = 47 (12.0%)** | **Survivors**  **N = 345 (88.0%)** | **P** |
| --- | --- | --- | --- | --- |
| **Demographics and chronic underlying conditions** | | | | |
| Male sex | 234 (59.7) | 22 (46.8) | 212 (61.4) | **0.028** |
| Age (median, IQR) | 65.0 (55.0-73.0) | 71.0 (65.0-77.0) | 63.0 (54.5-72.0) | **<0.001** |
| Age >70 years | 134 (34.2) | 26 (55.3) | 108 (31.3) | **0.001** |
| Smoking (>10 packets/year) | 61 (15.6) | 9 (19.1) | 52 (15.1) | 0.807 |
| Diabetes mellitus | 111 (28.4) | 17 (36.1) | 94 (27.3) | 0.322 |
| Chronic kidney disease | 112 (28.6) | 18 (38.3) | 94 (27.3) | 0.107 |
| Charlson Comorbidity Index ≥3 | 331 (84.4) | 43 (91.5) | 288 (83.5) | 0.427 |
| Chronic prednisone therapy (>10 mg/day | 114 (31.1) | 11 (28.9) | 103 (31.3) | 0.408 |
| **Type of immunocompromise** | | | | |
| Hematological disease | 134 (34.2) | 14 (10.4) ^1^ | 120 (89.6) ^1^ | 0.429 |
| Solid organ transplant | 178 (45.5) | 26 (14.6) ^1^ | 152 (85.4) ^1^ |  |
| Solid neoplasia | 58 (14.9) | 6 (10.3) ^1^ | 52 (89.7) ^1^ |  |
| Others | 22 (5.4) | 1 (4.5) ^1^ | 21 (95.5) ^1^ |  |
| ***Variant of Concern periods*** | | | | |
| Alpha | 50 (12.8) | 14 (28.0) ^2^ | 36 (72.0) ^2^ | **<0.001** |
| Delta | 36 (9.2) | 9 (25.0) ^2^ | 27 (75.0) ^2^ |  |
| Omicron | 306 (78.1) | 24 (7.8) ^2^ | 282 (91.5) ^2^ |  |
| ***COVID-19 vaccination in all patients and by VOC periods*** | | | | |
| All vaccinated patients | 338 (86.2) | 33 (70.2) | 305 (88.4) | **<0.001** |
| Alpha | 6 (1.8) | 1 (16.7) ^3^ | 5 (83.3) ^3^ | **0.013** |
| Delta | 34 (10.1) | 8 (23.5) ^3^ | 26 (76.5) ^3^ |  |
| Omicron | 298 (88.2) | 24 (8.1) ^3^ | 274 (91.9) ^3^ |  |
| **Symptoms and signs at diagnosis** | | | | |
| Fever | 94 (25.5) | 11 (23.4) | 83 (25.9) | 0.839 |
| Cough | 264 (67.5) | 33 (70.2) | 231 (67.2) | 0.095 |
| Dyspnea | 160 (40.8) | 31 (66.0) | 129 (37.4) | **0.022** |
| SpO_2_ <95% | 130 (33.3) | 22 (46.8) | 108 (31.5) | 0.603 |
| Pneumonia | 280 (71.4) | 42 (89.4) | 238 (69.0) | 0.522 |
| CURB-65 ≥2 | 63 (28.4) | 18 (51.4) | 45 (24.1) | **<0.001** |
| WHO clinical progression scale 6-9 ^4^ | 37 (9.4) | 12 (25.5) | 25 (7.2) | 0.140 |
| **Laboratory findings at diagnosis** | | | | |
| Neutrophil count >7500/μL | 69 (18.5) | 12 (26.7) | 57 (17.4) | 0.142 |
| Neutrophil count (median, IQR) (x10 00) | 4.2 (2.6-6.6) | 5.0 (2.9-8.2) | 4.2 (2.6-6.4) | 0.494 |
| Lymphocyte count<1000/µL | 260 (68.8) | 35 (74.5) | 225 (68.0) | 0.911 |
| Lymphocyte count (median, IQR) (x1000) | 0.7 (0.4-1.2) | 0.6 (0.4-1.1) | 0.7 (0.5-1.2) | 0.843 |
| Platelets <130 000/μL | 109 (28.8) | 9 (19.1) | 100 (30.2) | 0.123 |
| Platelets  (median, IQR) ) (x1000) | 166.5  (124.0-235.2) | 161.0  (137.0-199.0) | 169.0  (122.0-237.0) | 0.864 |
| Creatinine >1.3 mg/dL | 179 (47.4) | 28 (59.6) | 151 (45.6) | 0.221 |
| Creatinine, mg/dL (median [IQR]) | 1.3 (0.9-1.9) | 1.6 (1.1-2.2) | 1.2 (0.9-1.9) | 0.219 |
| C-reactive protein > 100 mg/L | 140 (37.4) | 23 (48.9) | 117 (35.8) | 0.372 |
| C-reactive protein mg/L  (median [IQR]) | 75.5  (32.7-135.1) | 99.2  (58.2-205.0) | 70.7  (29.9-132.2) | **0.006** |
| D-dimer >600 ng/mL | 199 (67.5) | 28 (71.8) | 171 (66.8) | 0.204 |
| D-dimer  (median, IQR) | 860.0  (520.0-1650.0) | 1058.0  (550.0-1750.0) | 845.0  (512.5-1613.5) | 0.055 |
| LDH >300 IU/L | 169 (48.7) | 30 (65.2) | 139 (46.2) | 0.499 |
| LDH IU/L  (median [IQR]) | 295.0  (227.0-377.0) | 332.0  (265.7-403.0) | 289.0  (223.5-371.5) | 0.605 |
| IgM positive | 80 (36.9) | 15 (51.7) | 65 (34.6) | **0.031** |
| IgM ng/ml  (median, IQR) | 77.4  (42.6-180.7) | 98.4  (57.2-159.1) | 74.1  (41.6-196.4) | 0.213 |
| IgG positive | 176 (82.2) | 24 (80.0) | 152 (82.6) | 0.715 |
| IgG ng/ml  (median, IQR) | 1532.0  (348.8-3180.9) | 1029.0  (219.0-3049.6) | 1553.6  (492.5-3195.8) | 0.565 |
| Neutralizing antibodies absence | 180 (50.7) | 33 (73.3) | 147 (47.4) | **0.036** |
| Neutralizing antibodies  (GMT, IC95) | 107.0  (79.0-146.1) | 56.9  (26.5-118.4) | 112.1  (81.2-159.3) | 0.425 |
| IFN-α undetectable | 32 (9.4) | 3 (7.0) | 29 (9.7) | 0.951 |
| IFN-α pg/mL (median, IQR) | 43.7 (19.6-81.5) | 29.7 (19.1-74.7) | 45.7 (19.6-83.1) | 0.317 |
| IFN-γ undetectable | 124 (35.0) | 17 (39.5) | 107 (34.4) | 0.516 |
| IFN-γ pg/mL (median, IQR) | 52.7  (9.8-84.9) | 76.8  (43.6-261.9) | 49.6  (9.2-77.8) | 0.146 |
| RNAemia | 195 (49.7) | 38 (80.9) | 157 (45.5) | **0.004** |
| **Treatment** | | | | |
| Antiviral | 179 (47.5) | 12 (31.6) | 167 (49.3) | **0.035** |
| Remdesivir | 118 (31.3) | 11 (28.9) | 107 (31.6) | 0.095 |
| Tocilizumab | 56 (14.9) | 17 (44.7) | 39 (11.5) | **0.008** |
| Dexamethasone | 216 (57.3) | 29 (70.7) | 187 (55.7) | 0.619 |
| Antibiotics | 97 (27.2) | 13 (36.1) | 84 (26.3) | 0.622 |
| **Outcome** | | | | |
| Hospital admission | 358 (91.3) | 47 (100) | 311 (90.1) | **-** |
| Length of hospital stay  (days, median [IQR]) | 8.0 (5.0-14.5) | 16.0 (10.0-21.0) | 8.0 (5.0-13.0) | **<0.001** |
| HFNO ^5^ | 24 (6.1) | 15 (31.9) | 9 (2.6) | **0.001** |
| Intensive care unit admission | 32 (8.2) | 13 (30.2) | 19 (5.5) | 0.314 |
| WHO clinical progression scale 7–10 ^4^ | 58 (14.8) | 47 (100) | 11 (3.2) | **<0.001** |

^1^ Percentages respect to the types of immunocompromise; ^2^ Percentages respect to the patients in each VOC period; ^3^ Percentages respect to the vaccinated patients in each VOC period; ^4^ <https://doi.org/10.1016/S1473-3099(20)30483-7>; ^5^ HFNO: High-Flow Nasal Oxygen.

**Table S4.** Demographics, chronic underlying diseases, clinical characteristics and outcomes of immunocompetent sub-cohort according to 30-day all-cause mortality.

| **Variables** | **Immunocompetent sub-cohort**  **N = 619** | **Dead**  **N = 32 (5.2%)** | **Survivors**  **N = 587 (94.8%)** | **P** |
| --- | --- | --- | --- | --- |
| **Demographics and chronic underlying conditions** | | | | |
| Male sex | 395 (63.8) | 17 (53.1) | 378 (64.4) | 0.069 |
| Age (median, IQR) | 64 (53-77) | 80 (68.5-84.5) | 63.0 (52.0-76.0) | **<0.001** |
| Age >70 years | 220 (35.5) | 23 (71.9) | 197 (33.6) | **<0.001** |
| Smoking (>10 packets/year) | 40 (6.9) | 1 (3.1) | 39 (7.1) | 0.698 |
| Diabetes mellitus | 165 (26.7) | 12 (37.5) | 153 (26.1) | 0.191 |
| Chronic kidney disease | 51 (8.3) | 4 (12.5) | 47 (8.0) | 0.606 |
| Charlson Comorbidity Index ≥3 | 342 (55.3) | 31 (96.9) | 311 (53.0) | **0.003** |
| Chronic prednisone therapy (>10 mg/day | 40 (8.3) | 7 (25.9) | 33 (7.3) | **0.001** |
| ***Variant of Concern periods*** | | | | |
| Alpha | 388 (62.7) | 17 (4.4) ^1^ | 371 (95.6) ^1^ | 0.302 |
| Delta | 160 (25.8) | 12 (7.5) ^1^ | 148 (92.5) ^1^ |  |
| Omicron | 71 (11.5) | 3 (4.2) ^1^ | 68 (95.8) ^1^ |  |
| ***COVID-19 vaccination in all patients and by VOC periods*** | | | | |
| All vaccinated patients | 220 (35.5) | 16 (50.0) | 204 (34.8) | 0.079 |
| Alpha | 9 (4.1) | 2 (22.2) ^2^ | 7 (77.8) ^2^ | 0.091 |
| Delta | 147 (66.8) | 12 (8.2) ^2^ | 135 (91.8) ^2^ |  |
| Omicron | 64 (29.1) | 2 (3.1) ^2^ | 62 (96.9) ^2^ |  |
| **Symptoms and signs at diagnosis** | | | | |
| Fever | 168 (27.1) | 10 (31.3) | 158 (26.9) | 0.990 |
| Cough | 411 (66.5) | 20 (62.5) | 391 (66.7) | 0.818 |
| Dyspnea | 324 (52.4) | 23 (71.9) | 301 (51.5) | 0.074 |
| SpO_2_ <95% | 247 (40.0) | 19 (59.4) | 228 (39.0) | 0.456 |
| Pneumonia | 539 (88.8) | 28 (87.5) | 511 (88.9) | 0.507 |
| CURB-65 ≥2 | 90 (21.3) | 16 (72.7) | 74 (18.5) | **<0.001** |
| WHO clinical progression scale 6-9 ^3^ | 40 (6.5) | 7 (21.9) | 33 (5.6) | 0.338 |
| **Laboratory findings at diagnosis** | | | | |
| Neutrophil count >7500/μL | 126 (21.0) | 7 (21.9) | 119 (20.9) | 0.644 |
| Neutrophil count (median, IQR) (x1000) | 4.7 (3.5-6.9) | 5.4 (3.3-7.3) | 4.7 (3.5-6.9) | 0.701 |
| Lymphocyte count<1000/µL | 286 (46.4) | 23 (71.9) | 263 (45.0) | 0.082 |
| Lymphocyte count (median, IQR) (x1000) | 1.0 (0.7-1.5) | 0.7 (0.6-1.2) | 1.1 (0.8-1.5) | 0.463 |
| Platelets <130 000/μL | 73 (11.8) | 5 (15.6) | 68 (11.6) | 0.835 |
| Platelets  (median, IQR) ) (x1000) | 206.0  (155.0-271.5) | 184.0  (145.5-256.7) | 208.0  (155.5-272.5) | 0.946 |
| Creatinine >1.3 mg/d | 101 (16.4) | 11 (34.4) | 90 (15.5) | 0.101 |
| Creatinine, mg/dL (median [IQR]) | 0.9 (0.7-1.1) | 1.2 (1.0-1.7) | 0.9 (0.7-1.1) | 0.304 |
| C-reactive protein > 100 mg/L | 204 (33.4) | 19 (59.4) | 185 (32.0) | **0.020** |
| C-reactive protein mg/L  (median [IQR]) | 63.4 (25.4-129.2) | 118.8 (60.5-195.8) | 59.7 (24.0-120.6) | **0.050** |
| D-dimer >600 ng/mL | 264 (48.5) | 17 (68.0) | 247 (47.6) | 0.097 |
| D-dimer  (median, IQR) | 570.0  (330.0-1077.5) | 872.0  (525.0-2500.0) | 570.0  (330.0-1050.0) | 0.343 |
| LDH >300 IU/L | 277 (46.0) | 18 (58.1) | 259 (45.4) | 0.457 |
| LDH IU/L  (median [IQR]) | 288.5  (236.0-365.0) | 365.0  (274.0-511.0) | 287.0  (233.0-358.0) | 0.108 |
| IgM positive | 258 (63.2) | 10 (58.8) | 248 (63.4) | 0.973 |
| IgM ng/ml  (median, IQR) | 94.5 (60.0-242.3) | 82.4 (72.1-347.1) | 96.4 (59.3-238.2) | 0.920 |
| IgG positive | 355 (86.6) | 14 (82.4) | 341 (86.8) | 0.966 |
| IgG ng/ml  (median, IQR) | 2279.0  (750.7-3621.3) | 2923.8  (1798.3-3555.1) | 2220.0  (731.1-3668.2) | 0.859 |
| Neutralizing antibodies absence | 52 (17.7) | 4 (20.0) | 48 (17.5) | 0.654 |
| Neutralizing antibodies  (GMT, IC95) | 359.4  (275.0-480.0) | 205.2  (70.9-546.9) | 373.9  (278.0-500.4) | 0.335 |
| IFN-α undetectable | 54 (13.3) | 3 (15.8) | 51 (13.2) | 0.594 |
| IFN-α pg/mL (median, IQR) | 18.3 (9.3-29.4) | 30.4 (20.1-88.3) | 18.0 (9.0-28.6) | **<0.001** |
| IFN-γ undetectable | 91 (18.3) | 7 (29.2) | 84 (17.7) | 0.216 |
| IFN-γ pg/mL (median, IQR) | 107.0  (25.0-272.9) | 88.0  (34.1-227.0) | 107.1  (24.1-279.24) | **0.032** |
| RNAemia | 116 (18.7) | 14 (43.8) | 102 (17.4) | 0.051 |
| **Treatment** | | | | |
| Antiviral | 90 (14.6) | 4 (12.5) | 86 (14.7) | 0.334 |
| Remdesivir | 90 (14.6) | 4 (12.5) | 86 (14.7) | 0.334 |
| Tocilizumab | 67 (10.8) | 8 (25.0) | 59 (10.1) | 0.665 |
| Dexamethasone | 342 (65.6) | 22 (81.5) | 320 (64.8) | 0.090 |
| Antibiotics | 83 (15.3) | 8 (33.3) | 75 (14.5) | 0.194 |
| **Outcome** | | | | |
| Hospital admission | 611 (98.7) | 32 (100) | 579 (98.6) | **-** |
| Length of hospital stay  (days, median [IQR]) | 6.0 (4.0-10.0) | 9.0 (5.0-15.75) | 7.0 (4.0-10.0) | **<0.001** |
| HFNO ^4^ | 24 (3.9) | 4 (12.5) | 20 (3.4) | **0.417** |
| Intensive care unit admission | 44 (7.1) | 4 (12.5) | 40 (6.8) | 0.056 |
| WHO clinical progression scale 7–10 ^3^ | 71 (11.5) | 32 (100) | 39 (6.6) | **0.001** |

^1^ Percentages respect to the patients in each VOC period; ^2^ Percentages respect to the vaccinated patients in each VOC period; ^3^ <https://doi.org/10.1016/S1473-3099(20)30483-7>; ^4^ HFNO: High-Flow Nasal Oxygen.

**Table S5.** Patients treated with remdesivir, dexamethasone or tocilizumab according the WHO clinical progression scale (WHO-CPS)^1^ at COVID-19 diagnosis in the whole cohort and in the immunocompromised and immunocompetent sub-cohorts.

|  | **Whole cohort**  **N = 1011** | **WHO-CPS 6-9**  **N = 77 (7.6%)** | **WHO-CPS 2-5**  **N = 934 (92.4%)** | **P** |
| --- | --- | --- | --- | --- |
| Remdesivir | 208 (20.9) | 16 (21.1) | 192 (20.9) | 0.960 |
| Dexamethasone | 558 (62.1) | 64 (88.9) | 494 (59.8) | **<0.001** |
| Tocilizumab | 123 (12.4) | 47 (61.8) | 76 (8.3) | **<0.001** |
|  | **Immunocompromised sub-cohort N = 392** | **WHO-CPS 6-9**  **N = 37 (9.4%)** | **WHO-CPS 2-5**  **N = 355 (90.6%)** | **P** |
| Remdesivir | 118 (31.3) | 12 (33.3) | 106 (31.1) | 0.745 |
| Dexamethasone | 216 (57.3) | 32 (88.9) | 184 (54.0) | **<0.001** |
| Tocilizumab | 56 (14.9) | 24 (66.7) | 32 (9.4) | **<0.001** |
|  | **Immunocompetent sub-cohort N = 619** | **WHO-CPS 6-9**  **N = 40 (6.5%)** | **WHO-CPS 2-5**  **N = 579 (93.5%)** | **P** |
| Remdesivir | 90 (14.6) | 4 (10.0) | 86 (14.9) | 0.398 |
| Dexamethasone | 342 (65.6) | 32 (88.9) | 310 (63.9) | **0.002** |
| Tocilizumab | 67 (10.8) | 23 (57.5) | 44 (7.6) | **<0.001** |

^1^ <https://doi.org/10.1016/S1473-3099(20)30483-7>.

**Table S6.** Demographics, chronic underlying diseases, clinical characteristics and outcomes of immunocompromised sub-cohort with *vs.* without SARS-CoV-2 RNAemia at COVID-19 diagnosis.

| **Variables** | **Immunocompromised**  **Sub-cohort N = 392** | **RNAemia**  **N = 195 (49.7%)** | **No RNAemia**  **N = 197 (50.3%)** | **P** |
| --- | --- | --- | --- | --- |
| **Demographics and chronic underlying conditions** | | | | |
| Male sex | 234 (59.7) | 124 (63.6) | 110 (55.8) | 0.118 |
| Age (median, IQR) | 65.0 (55.0-73.0) | 66 (57.0-73.0) | 63.0 (51.5-73.0) | **0.018** |
| Age >70 years | 134 (34.2) | 71 (36.4) | 63 (32.0) | 0.355 |
| Smoking (>10 packets/year) | 61 (15.6) | 33 (17.0) | 28 (14.2) | 0.446 |
| Diabetes mellitus | 111 (28.3) | 64 (32.8) | 47 (23.9) | **0.049** |
| Chronic kidney disease | 112 (28.6) | 62 (32.0) | 50 (25.4) | 0.150 |
| Charlson Comorbidity Index ≥3 | 331 (84.4) | 171 (87.7) | 160 (81.2) | 0.077 |
| Chronic prednisone therapy (>10 mg/day) | 114 (31.1) | 58 (31.5) | 56 (30.6) | 0.849 |
| **Type of immunocompromise** | | | | |
| Hematological disease | 134 (34.2) | 75 (56.0) ^1^ | 59 (44.0) ^1^ | **0.045** |
| Solid organ transplant | 178 (45.5) | 91 (51.1) ^1^ | 87 (48.9) ^1^ |  |
| Solid neoplasia | 58 (14.9) | 21 (36.2) ^1^ | 37 (63.8) ^1^ |  |
| Others | 22 (5.4) | 8 (36.4) ^1^ | 14 (63.6) ^1^ |  |
| ***Variant of Concern periods*** | | | | |
| Alpha | 50 (12.8) | 30 (60.0) ^2^ | 20 (40.0) ^2^ | **0.050** |
| Delta | 36 (9.2) | 12 (33.3) ^2^ | 24 (66.7) ^2^ |  |
| Omicron | 306 (78.1) | 153 (50.0) ^2^ | 153 (50.0) ^2^ |  |
| ***COVID-19 vaccination in all patients and by VOC periods*** | | | | |
| All vaccinated patients | 338 (86.2) | 164 (84.1) | 174 (88.3) | 0.225 |
| Alpha | 6 (1.8) | 4 (66.7) ^3^ | 2 (33.3) ^3^ | 0.099 |
| Delta | 34 (10.1) | 11 (32.3) ^3^ | 23 (67.7) ^3^ |  |
| Omicron | 298 (88.2) | 149 (50.0) ^3^ | 149 (50.0) ^3^ |  |
| **Symptoms and signs at diagnosis** | | | | |
| Fever | 94 (25.5) | 49 (26.2) | 45 (24.9) | 0.768 |
| Cough | 264 (67.5) | 137 (70.6) | 127 (64.5) | 0.194 |
| Dyspnea | 160 (40.8) | 87 (44.6) | 73 (37.1) | 0.128 |
| SpO_2_ <95% | 130 (33.3) | 80 (41.0) | 50 (25.6) | **0.001** |
| Pneumonia | 280 (71.4) | 153 (78.5) | 127 (64.5) | **0.002** |
| CURB-65 ≥2 | 63 (28.4) | 36 (28.8) | 27 (27.8) | 0.874 |
| WHO clinical progression scale 6-9 ^4^ | 37 (9.4) | 28 (14.4) | 9 (4.6) | **0.001** |
| **Laboratory findings at diagnosis** | | | | |
| Neutrophil count >7500/μL | 69 (18.5) | 34 (18.0) | 35 (19.0) | 0.797 |
| Neutrophil count (median, IQR) (x10 00) | 4.2 (2.6-6.6) | 4.0 (2.5-6.6) | 4.6 (2.7.6.5) | 0.322 |
| Lymphocyte count<1000/µL | 260 (68.8) | 140 (72.9) | 120 (64.5) | 0.078 |
| Lymphocyte count (median, IQR) (x1000) | 0.7 (0.4-1.2) | 0.6 (0.4-1.1) | 0.8 (0.5-1.3) | **0.035** |
| Platelets <130 000/μL | 109 (28.8) | 57 (29.7) | 52 (28.0) | 0.710 |
| Platelets  (median, IQR) ) (x1000) | 166.5  (124.0-235.2) | 161.0  (122.5-228.0) | 176.0  (124.7-239.2) | 0.250 |
| Creatinine >1.3 mg/dL | 179 (47.4) | 100 (52.1) | 79 (42.5) | 0.061 |
| Creatinine, mg/dL (median [IQR]) | 1.3 (0.9-1.9) | 1.3 (0.9-2.1) | 1.2 (0.8-1.9) | **0.023** |
| C-reactive protein > 100 mg/L | 140 (37.4) | 83 (43.5) | 57 (31.1) | **0.014** |
| C-reactive protein mg/L  (median [IQR]) | 75.5  (32.7-135.1) | 89.9  (45.6-145.1) | 62.3  (21.0-120.3) | **<0.001** |
| D-dimer >600 ng/mL | 199 (67.5) | 107 (70.4) | 92 (64.3) | 0.267 |
| D-dimer  (median, IQR) | 860.0  (520.0-1650.0) | 915.0  (532.5-1700.0) | 820.0  (480.0-1540.0) | 0.152 |
| LDH >300 IU/L | 169 (48.7) | 101 (55.8) | 68 (41.0) | **0.006** |
| LDH IU/L  (median [IQR]) | 295.0  (227.0-377.0) | 317.0  (232.5-413.0) | 276.0  (223.0-344.0) | **0.018** |
| IgM positive | 80 (36.9) | 34 (29.8) | 46 (44.7) | **0.024** |
| IgM ng/ml  (median, IQR) | 77.4  (42.6-180.7) | 95.0  (41.2-196.6) | 75.1  (44.1-161.1) | 0.827 |
| IgG positive | 176 (82.2) | 91 (80.5) | 85 (84.2) | 0.488 |
| IgG ng/ml  (median, IQR) | 1532.0  (348.8-3180.9) | 1278.1  (252.3-2954.1) | 2127.8  (819.8-5466.3) | **0.006** |
| Neutralizing antibodies absence | 180 (50.7) | 114 (63.3) | 66 (37.7) | **<0.001** |
| Neutralizing antibodies  (GMT, IC95) | 107.0  (79.0-146.1) | 65.6  (44.3-97.3) | 143.9  (93.8-229.2) | **0.046** |
| IFN-α undetectable | 32 (9.4) | 12 (6.8) | 20 (12.0) | 0.097 |
| IFN-α pg/mL (median, IQR) | 43.70  (19.59-81.50) | 45.08  (21.11-81.79) | 42.37  (16.95-77.90) | 0.501 |
| IFN-γ undetectable | 124 (35.0) | 67 (37.2) | 57 (32.8) | 0.379 |
| IFN-γ pg/mL (median, IQR) | 52.74  (9.84-84.90) | 52.50  (14.42-89.41) | 53.79  (9.20-78.64) | 0.708 |
| **Treatment** | | | | |
| Antiviral | 179 (47.5) | 98 (53.0) | 81 (42.2) | **0.036** |
| Remdesivir | 118 (31.3) | 75 (40.5) | 43 (22.4) | **<0.001** |
| Tocilizumab | 56 (14.9) | 44 (23.8) | 12 (6.3) | **<0.001** |
| Dexamethasone | 216 (57.3) | 128 (68.8) | 88 (46.1) | **<0.001** |
| Antibiotics | 97 (27.2) | 55 (32.2) | 42 (22.7) | **0.045** |
| **Outcome** | | | | |
| Hospital admission | 358 (91.3) | 184 (94.4) | 174 (88.3) | **0.034** |
| Length of hospital stay  (days, median [IQR]) | 8.0 (5.0-14.5) | 10.0 (6.0-16.75) | 6.0 (4.0-13.0) | **<0.001** |
| HFNO ^5^ | 24 (6.1) | 17 (8.7) | 7 (3.6) | **0.033** |
| Intensive care unit admission | 32 (8.2) | 22 (11.4) | 10 (5.1) | **0.025** |
| WHO clinical progression scale 7–10 ^4^ | 58 (14.8) | 45 (23.1) | 13 (6.6) | **<0.001** |
| Mortality at day +30 | 47 (12.0) | 38 (19.5) | 9 (4.6) | **<0.001** |

^1^ Percentages respect to the types of immunocompromise; ^2^ Percentages respect to the patients in each VOC period; ^3^ Percentages respect to the vaccinated patients in each VOC period; ^4^ <https://doi.org/10.1016/S1473-3099(20)30483-7>; ^5^ HFNO: High-Flow Nasal Oxygen.

**Table S7.** Risk factors associated with the presence of RNAemia in the different types of immunocompromise in the immunocompromised sub-cohort: Multivariate logistic regression analyses.

| **Hematological malignancies** | | |
| --- | --- | --- |
| ***Variables - Model A (N = 118)*** | **OR (95% CI)** | **P** |
| Alpha and Omicron periods | 0.73 (0.12-4.38) | 0.727 |
| Pneumonia | 2.45 (1.03-5.83) | **0.043** |
| Neutralizing antibodies absence | 1.62 (0.72-3.62) | 0.239 |
|  | | |
| ***Variables - Model B (N = 118)*** | **OR (95% CI)** | **P** |
| Pneumonia | 2.47 (1.04-5.87) | **0.041** |
| Neutralizing antibodies absence | 1.62 (0.72-3.62) | 0.239 |
|  | | |
| ***Variables - Model C (N = 134)*** | **OR (95% CI)** | **P** |
| Pneumonia | 2.53 (1.18-5.40) | **0.016** |
| **Solid organ transplantation** | | |
| ***Variables – Model A (N = 167)*** | **OR (95% CI)** | **P** |
| Alpha and Omicron periods | 2.31 (0.83-6.43) | 0.109 |
| Pneumonia | 2.53 (1.15-5.54) | **0.021** |
| Neutralizing antibodies absence | 2.79 (1.46-5.32) | **0.002** |
|  | | |
| ***Variables – Model B (N = 167)*** | **OR (95% CI)** | **P** |
| Pneumonia | 2.50 (1.15-5.47) | **0.021** |
| Neutralizing antibodies absence | 2.81 (1.48-5.35) | **0.002** |
| **Solid neoplasia** | | |
| ***Variables – Model B (N = 47)*** | **OR (95% CI)** | **P** |
| Alpha and Omicron periods | 1.43 (0.38-5.36) | 0.602 |
| LDH >300 IU/L | 3.47 (0.88-13.65) | 0.075 |
| Neutralizing antibodies absence | 5.47 (1.32-22.55) | **0.019** |
|  | | |
| ***Variables – Model B (N = 47)*** | **OR (95% CI)** | **P** |
| LDH >300 IU/L | 3.42 (0.87-13.39) | 0.078 |
| Neutralizing antibodies absence | 5.37 (1.32-21.87) | **0.019** |

**Table S8.** Demographics, chronic underlying diseases, clinical characteristics and outcomes of immunocompetent sub-cohort with *vs.* without SARS-CoV-2 RNAemia at COVID-19 diagnosis.

| **Variables** | **Immunocompetent**  **Sub-cohort N = 619** | **RNAemia**  **N =116 (18.7%)** | **No RNAemia**  **N = 503 (81.3%)** | **P** |
| --- | --- | --- | --- | --- |
| **Demographics and chronic underlying conditions** | | | | |
| Male sex | 395 (63.8) | 76 (65.5) | 319 (63.4) | 0.672 |
| Age (median, IQR) | 64 (53-77) | 66 (55-76) | 63 (52-77) | 0.516 |
| Age >70 years | 220 (35.5) | 40 (34.5) | 180 (35.8) | 0.792 |
| Smoking (>10 packets/year | 40 (6.9) | 5 (4.4) | 35 (7.5) | 0.238 |
| Diabetes mellitus | 165 (26.7) | 29 (25.0) | 136 (27.0) | 0.655 |
| Chronic kidney disease | 51 (8.3) | 11 (9.6) | 40 (8.0) | 0.571 |
| Charlson Comorbidity Index ≥3 | 342 (55.3) | 66 (56.9) | 276 (54.9) | 0.692 |
| Chronic prednisone therapy (>10 mg/day | 40 (8.3) | 13 (13.4) | 27 (7.0) | **0.043** |
| ***Variant of Concern periods*** | | | | |
| Alpha | 388 (62.7) | 57 (14.7) ^1^ | 331 (85.3) ^1^ | **0.003** |
| Delta | 160 (25.8) | 40 (25.0) ^1^ | 120 (75.0) ^1^ |  |
| Omicron | 71 (11.5) | 19 (26.8) ^1^ | 52 (73.2) ^1^ |  |
| ***COVID-19 vaccination in all patients and by VOC periods*** | | | | |
| All vaccinated patients | 220 (35.5) | 56 (48.3) | 164 (32.6) | **0.001** |
| Alpha | 9 (4.1) | 1 (11.1) ^2^ | 8 (88.9) ^2^ | 0.597 |
| Delta | 147 (66.8) | 38 (25.9) ^2^ | 109 (74.1) ^2^ |  |
| Omicron | 64 (29.1) | 17 (26.6) ^2^ | 47 (73.4) ^2^ |  |
| **Symptoms and signs at diagnosis** | | | | |
| Fever | 168 (27.1) | 40 (34.5) | 128 (25.4) | **0.049** |
| Cough | 411 (66.5) | 87 (75.0) | 324 (64.5) | **0.031** |
| Dyspnea | 324 (52.4) | 76 (65.5) | 248 (49.5) | **0.002** |
| SpO_2_ <95% | 247 (40.0) | 62 (53.4) | 185 (36.9) | **0.001** |
| Pneumonia | 539 (88.8) | 105 (93.8) | 434 (87.7) | 0.066 |
| CURB-65 ≥2 | 90 (21.3) | 28 (33.7) | 62 (18.3) | **0.002** |
| WHO clinical progression scale 6-9 ^3^ | 40 (6.5) | 18 (15.5) | 22 (4.4) | **<0.001** |
| **Laboratory findings at diagnosis** | | | | |
| Neutrophil count >7500/μL | 126 (21.0) | 32 (28.6) | 94 (19.2) | **0.028** |
| Neutrophil count (median, IQR) (x1000) | 4.7 (3.5-6.9) | 5.1 (3.5-7.8) | 4.7 (3.4-6.7) | 0.130 |
| Lymphocyte count<1000/µL | 286 (46.4) | 71 (61.2) | 215 (42.9) | **<0.001** |
| Lymphocyte count (median, IQR) (x1000) | 1.0 (0.7-1.5) | 0.9 (0.6-1.3) | 1.1 (0.8-1.5) | **0.001** |
| Platelets <130 000/μL | 73 (11.8) | 18 (15.5) | 55 (11.0) | 0.173 |
| Platelets  (median, IQR) ) (x1000) | 206.0  (155.0-271.5) | 185.0  (152.0-255.7) | 213.0  (156.5-277.5) | **0.022** |
| Creatinine >1.3 mg/dL | 101 (16.4) | 26 (22.8) | 75 (15.0) | **0.042** |
| Creatinine, mg/dL (median [IQR]) | 0.9 (0.7-1.1) | 0.9 (0.7-1.3) | 0.9 (0.7-1.1) | **0.022** |
| C-reactive protein > 100 mg/L | 204 (33.4) | 62 (54.9) | 142 (28.6) | **<0.001** |
| C-reactive protein mg/L  (median [IQR]) | 63.4  (25.4-129.2) | 109.3  (50.5-179.0) | 54.0  (22.9-110.5) | **<0.001** |
| D-dimer >600 ng/mL | 264 (48.5) | 50 (52.1) | 214 (47.8) | 0.443 |
| D-dimer  (median, IQR) | 570.0  (330.0-1077.5) | 620.0  (368.5-973.7) | 570.0  (316.2-1080.0) | 0.482 |
| LDH >300 IU/L | 277 (46.0) | 81 (72.3) | 196 (40.0) | **<0.001** |
| LDH IU/L  (median [IQR]) | 288.5  (236.0-365.0) | 348.5  (281.0-480.2) | 276.0  (228.7-344.0) | **<0.001** |
| IgM positive | 258 (63.2) | 51 (60.7) | 207 (63.9) | 0.591 |
| IgM ng/ml  (median, IQR) | 94.5  (60.0-242.3) | 80.4  (57.1-181.5) | 96.9  (62.2-255.2) | 0.394 |
| IgG positive | 355 (86.6) | 73 (86.9) | 282 (86.5) | 0.923 |
| IgG ng/ml  (median, IQR) | 2279.0  (750.7-3621.3) | 2786.0  (863.8-3744.8) | 2130.0  (687.4-3612.7) | 0.184 |
| Neutralizing antibodies absence | 52 (17.7) | 14 (19.7) | 38 (17.0) | 0.607 |
| Neutralizing antibodies  (GMT, IC95) | 359.4  (275.0-480.0) | 340.3  (193.5-584.9) | 365.5  (265.4-511.5) | 0.794 |
| IFN-α undetectable | 54 (13.3) | 12 (17.1) | 42 (12.5) | 0.298 |
| IFN-α pg/mL (median, IQR) | 18.32  (9.28-29.43) | 23.23  (9.60-50.80) | 17.65  (8.93-25.27) | **0.014** |
| IFN-γ undetectable | 91 (18.3) | 13 (13.5) | 78 (19.4) | 0.182 |
| IFN-γ pg/mL (median, IQR) | 106.97  (24.96-272.88) | 94.00  (12.72-176.19) | 107.08  (27.99-355.20) | 0.119 |
| **Treatment** | | | | |
| Antiviral | 90 (14.6) | 15 (13.0) | 75 (14.9) | 0.609 |
| Remdesivir | 90 (14.6) | 15 (13.0) | 75 (14.9) | 0.609 |
| Tocilizumab | 67 (10.8) | 33 (28.7) | 34 (6.8) | **<0.001** |
| Dexamethasone | 342 (65.6) | 86 (83.5) | 256 (61.2) | **<0.001** |
| Antibiotics | 83 (15.3) | 22 (21.0) | 61 (13.9) | 0.072 |
| **Outcome** | | | | |
| Hospital admission | 611 (98.7) | 115 (99.1) | 496 (98.6) | 0.649 |
| Length of hospital stay  (days, median [IQR] | 6 (4-10) | 8 (4-14) | 6 (4-9) | **0.001** |
| HFNO ^4^ | 24 (3.9) | 14 (12.1) | 10 (2.0) | **<0.001** |
| Intensive care unit admission | 44 (7.1) | 20 (17.2) | 24 (4.8) | **<0.001** |
| WHO clinical progression scale 7–10 ^3^ | 71 (11.5) | 30 (25.9) | 41 (8.2) | **<0.001** |
| Mortality at day +30 | 32 (5.2) | 14 (12.1) | 18 (3.6) | **<0.001** |

^1^ Percentages respect to the patients in each VOC period; ^2^ Percentages respect to the vaccinated patients in each VOC period; ^3^ <https://doi.org/10.1016/S1473-3099(20)30483-7>; ^4^ HFNO: High-Flow Nasal Oxygen.

**Figure S1.** Area under the receiver operating-characteristic curves (AUC-ROC) of the multiple regression analysis models for SARS-CoV-2 RNAemia.

1. **Whole cohort**

***Model A. AUC-ROC = 0.763 (standard error 0.016, 95% CI 0.731–0.795; p < 0.001)***


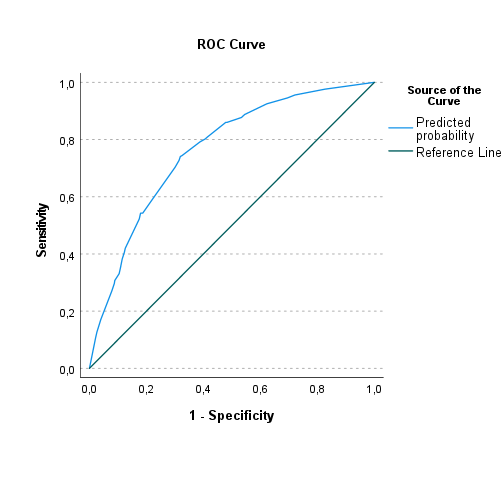


***Model B. AUC-ROC = 0.759 (standard error 0.016, 95% CI 0.726–0.791; p < 0.001)***


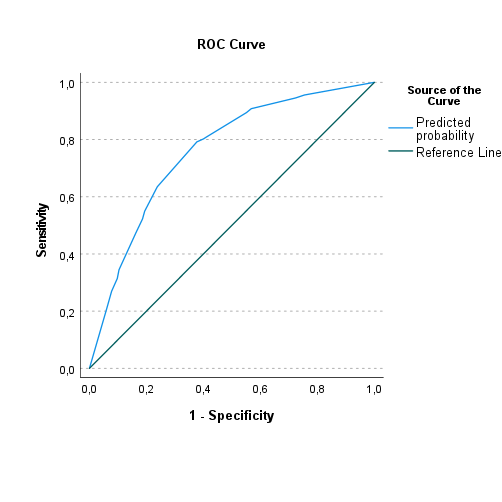


1. ***Immunocompromised sub-cohort***

***ROC/AUC = 0.678 (standard error 0.030, 95% CI 0.619–0.737; p < 0.001)***


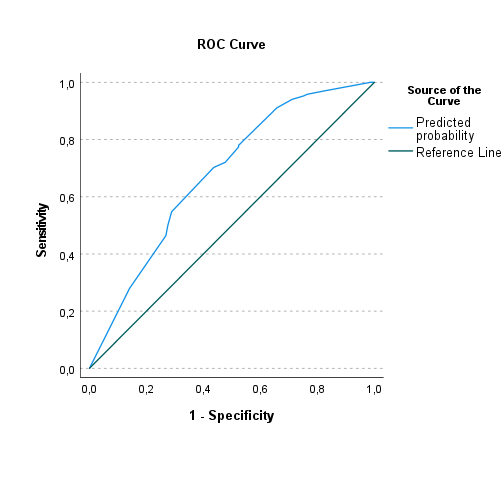


1. ***Immunocompetent sub-cohort***

***ROC/AUC = 0.720 (standard error 0.026, 95% CI 0.670–0.770; p < 0.001)***


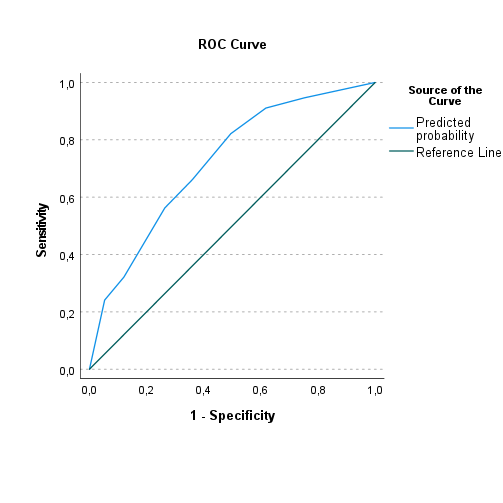

Supplement: S1 — (DOCX) [file pone.0330495.s001.docx]
